# miRNA Signature of Urine Extracellular Vesicles Shows the Involvement of Inflammatory and Apoptotic Processes in Diabetic Chronic Kidney Disease

**DOI:** 10.1007/s11095-023-03481-5

**Published:** 2023-03-01

**Authors:** Barbara Zapała, Agnieszka Kamińska, Monika Piwowar, Agnieszka Paziewska, Agnieszka Gala-Błądzińska, Ewa Ł. Stępień

**Affiliations:** 1 Department of Clinical Biochemistry, Jagiellonian University Medical College, Kraków, Poland; 2 Department of Medical Physics, M. Smoluchowski Institute of Physics, Faculty of Physics, Astronomy and Applied Computer Science, Jagiellonian University, Kraków, Poland; 3 Department of Bioinformatics and Telemedicine, Jagiellonian University Medical College, Kraków, Poland; 4 Department of Neuroendocrinology, Centre of Postgraduate Medical Education, Warszawa, Poland; 5 Institute of Health Sciences, Faculty of Medical and Health Sciences, Siedlce University of Natural Sciences and Humanities, Siedlce, Poland; 6 Medical College of Rzeszow University, Institute of Medical Sciences, Rzeszów, Poland; 7 Department of Internal Medicine, Nephrology and Endocrinology, St. Queen Jadwiga Clinical District Hospital No2 in Rzeszów, Rzeszów, Poland; 8 Total-Body Jagiellonian-PET Laboratory, Jagiellonian University, Kraków, Poland; 9 Center for Theranostics, Jagiellonian University, Kraków, Poland

**Keywords:** biomarkers, diabetic kidney disease, extracellular vesicles, micro-RNA, type 2 diabetes mellitus

## Abstract

**Background:**

The aim of this study was to investigate the role of urine-derived extracellular vesicles (uEVs) in diabetic kidney disease (DKD) in patients diagnosed with type 2 diabetes mellitus (T2DM).

**Methods:**

UEVs were characterized by size distribution and microRNA content by next-generation small RNA sequencing and quantitative reverse transcription PCR.

**Results:**

A subset of sixteen miRNAs enriched in T2DM patients with DKD, including hsa-miR-514a-5p, hsa-miR‑451a, hsa-miR-126-3p, hsa-miR-214, or hsa-miR‑503 was identified. Eight miRNAs as hsa-miR-21-3p, hsa-miR-4792, hsa-miR‑375, hsa-miR-1268a, hsa-miR-501-5p, or hsa-miR-582 were downregulated. Prediction of potential target genes and pathway enrichment analysis of the Kyoto Encyclopedia of Genes and Genomes (KEGG) confirmed possible functions related to cellular processes such as apoptosis, inflammation, and tissue remodeling, that promote diabetic complications, such as DKD. Among them, hsa-miR-375, hsa-miR-503, and hsa-miR-451a make important contribution. Additionally, downregulated hsa-miR-582-5p has not been reported so far in any diabetes-related pathways.

**Conclusions:**

This study revealed the most significant miRNAs in uEVs of patients with T2DM. However, as this is a bioinformatic prediction that we performed based on the putative targets of the identified miRNAs. Thus, further *in vitro* functional studies are needed to confirm our findings. Knowing the fact that EVs are crucial in transferring miRNAs, there is a great need toto discover their involvement in the pathomechanism of T2DM-related kidney disease.

**Supplementary Information:**

The online version contains supplementary material available at 10.1007/s11095-023-03481-5.

## Introduction

Type 2 diabetes mellitus (T2DM) affects more than 422 million people worldwide (2014) and is thought to be the second primary global disease of the twenty-first century [1]. Among several complications caused by T2DM, chronic kidney disease (CKD) is the most common complication in treating more than one-third of diabetic patients. It increases the mortality risk in patients with T2DM [2, 3]. Although T2DM and arterial hypertension are primarily involved in the development of CKD, diabetes, and hypertension are highly interlinked, and they are associated with alterations in the structure of the kidney, such as thickening of the glomerular basement membrane, loss of endothelial fenestrations, mesangial matrix expansion, and loss of podocytes [3]. For that reason, CDK related to diabetes is defined as diabetic kidney disease (DKD) and becomes end-stage kidney disease (ESRD) in about 50% of all patients with DKD after several decades of diabetes [4].

Urine is a valuable diagnostic medium commonly used to diagnose kidney diseases and many others. Proteomic analyzes of human urine revealed that a variety of membrane proteins were present in the ultracentrifuged or precipitated urine pellet, especially those related to cellular transport and signaling [5]. This groundbreaking discovery gave rise to the idea that new diagnostic indicators can be detected in a urine sediment, among them extracellular vesicles (EVs) proved useful for biomarkers investigation [6]. EVs are cell-derived membrane-capsulated particles, canonically subdivided into three classes of vesicles: exosomes, microvesicles, and apoptotic bodies. The subtypes of EVs differ in their size (ranging from 30 to 5000 nm) and in the mechanism of their biogenesis and function [7, 8]. It has been broadly accepted that EVs may be involved in both physiological and pathological conditions where they play a role in cellular communication by transferring membrane-derived receptors, and proteins such as cytokines, chemokines, glycans, and lipids [9–13].

EVs are supposedly responsible for removing unwanted molecular material, cellular waste, or disordered proteins [14, 15]. EVs are also capable of carrying genetic material, including mRNA and miRNAs [16–19]. EV-transferred miRNAs can play an essential role in cellular and biological processes such as proliferation, differentiation, apoptosis, stress resistance, angiogenesis, tissue remodeling, and many other cellular processes [15, 19–21]. There is growing evidence that EVs, that are enriched in biofluids such as blood or urine, are capable of carrying miRNAs and other biomolecules that play a vital role in kidney signaling by stimulating cells on the surface of vascular the compartment and cells of the immune system to contribute in kidney diseases [21, 22]. Prior reports have supported the idea that EVs are involved in intra-organ signaling, especially proximal-to-distal signaling, where EVs can be takenup downstream, affecting of recipient cell function. It has been shown that EVs from collecting duct cells can transfer functional aquaporin 2 (AQP2) into recipient cells [23].

Numerous kidney pathologies, such as glomerular and tubular damage, are associated with EV release due to DKD in T2DMpatients. Urine collected EVs (uEVs) may be very important for understanding kidney metabolism and pathological mechanisms, as uEVs can carry a variety of metabolic compounds and disease-specific biomarkers [24].

Our study focused on uEVs in the pathogenesis of diabetes-related chronic kidney dysfunction. After the procedure of uEVs isolation, we performed the next-generation sequencing (NGS) to characterize miRNAs enriched in the uEVs subsets from patients suffering from T2DM and affected by DKD. The results were compared with those of healthy individuals. This report showed that uEVs, which have an excellent potential for stratification of DKD patient, are enriched with unique miRNA subsets that regulate cell proliferation, migration, apoptosis, and inflammation, and are depleted with miRNAs-regulated metabolic processes related to the activity of insulin.

This study revealed the most significant miRNAs in uEVs of patients with T2DM. However, as this it is a bioinformatic prediction that we made based on the putative targets of the identified miRNAs. Therefore, further *in vitro* functional studies are needed to confirm our findings. Knowing that EVS are crucial in transferring of miRNAs, it is very important to discover their involvement in the pathomechanism of T2DM-related CKD.

## Results

### Study Group

The study was conducted on eight diabetic patients diagnosed with DKD. The control group consisted of six individuals. The distribution of selected epidemic risk factors among patients and controls is shown in Table I. Taking into account the distributions of these features, patients with DKD and healthy individuals did not differ significantly in terms of age, CHOL, LDL-CHOL and HDL-CHOL, and TG. HbA1c and serum creatinine levels and hs-CRP were higher in DKD patients compared to controls. T2DM patients showed decreasing eGFR.

**Table I:** Characteristics of the Study and Control Groups

	Control N = 6	Diabetes N = 8	p
Age (years)	50 (47 – 66)	82.5 (45 – 88)	0.307
Serum glucose (mmol/l)	5.3 (4.9 – 5.4)	13.6 (7.7 – 19.7)	**0.009**
HbA1c (%)	5 (4.7 – 5.0)	10.1 (7.3 – 14.0)	**0.002**
Serum creatinine (µmol/l)	66 32 – 89)	100 (88–120)	**0.058**
eGFR (ml/min/1.73cm^2^)	94 (81–137)	52 (35 – 81)	**0.043**
hsCRP (mg/ml)	0.5 (0.3 – 0.7)	12.5 (3.3–31.9)	**0.006**
CHOL (mmol/l)	5.1 (4.1 – 6.6)	4.6 (2.6 – 7.5)	0.533
LDL-CHOL (mmol/l)	2.9 (2.6 – 4.8)	2.0 (1.4 – 3.2)	0.309
HDL-CHOL (mmol/l)	1.3 (1.3 – 1.6)	1.0 (0.8 – 1.4)	0.127
TG (mmol/l)	1.0 (0.4 – 1.1)	1.1 (0.7 – 3.1)	0.447

Data are presented as medians and interquartile range. Analysis was performed using the nonparametric U-Mann Whitney test. Bold means statistically significant difference between groups

Abbreviations: HbA1c, glycated hemoglobin; EGFR, estimated Glomerular Filtration Rate; HsCRP, high sensitivity C-reactive protein; CHOL, cholesterol; LDL-CHOL, low-density lipoprotein cholesterol; HDL-CHOL, high-density lipoprotein cholesterol; TG, triglycerides

### Urinary EVs Characterization by Nanoparticle Tracking Analysis and Electron Microscopy

**Fig. 1:**
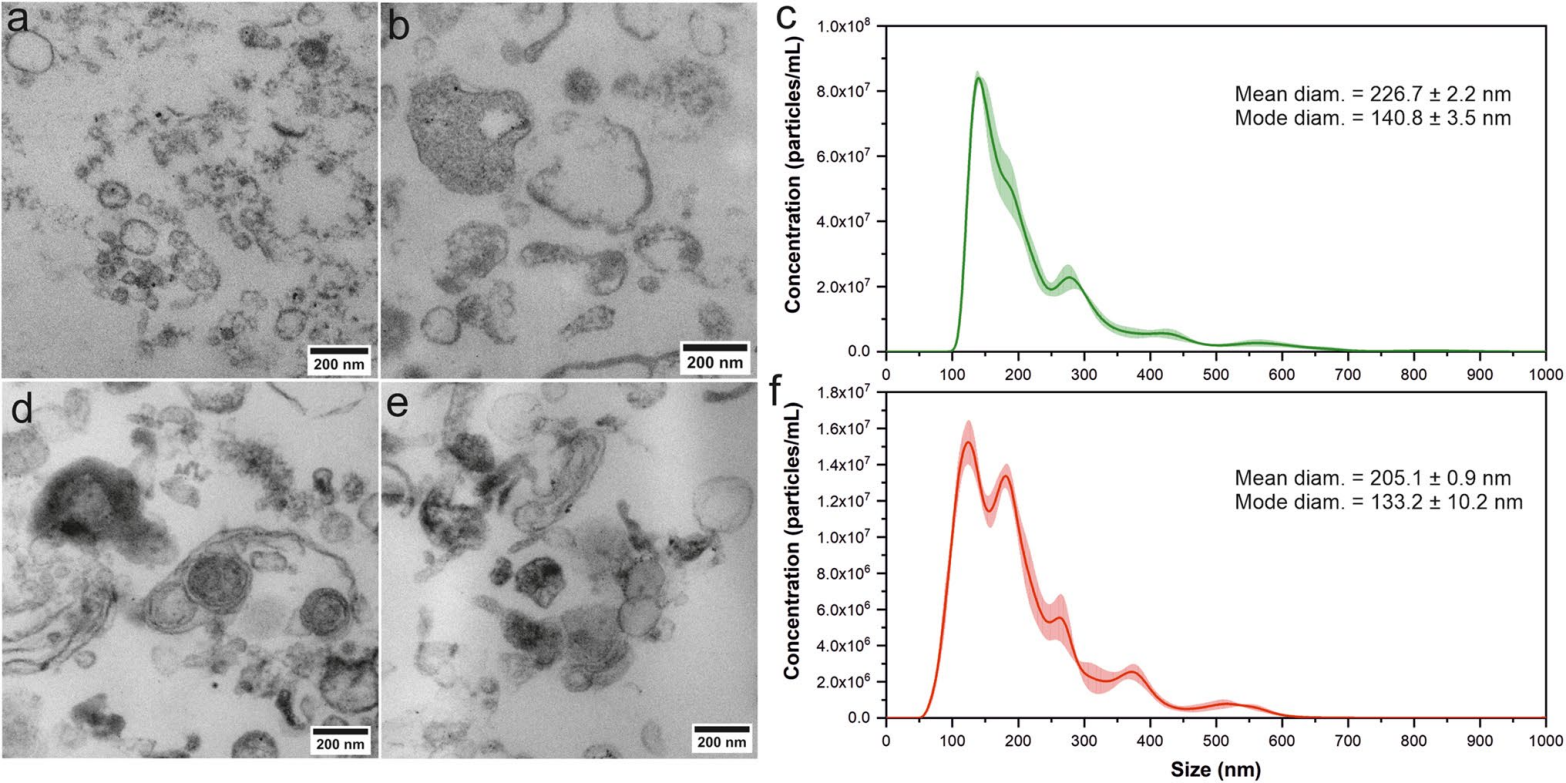
Representative electron microscopy images of uEVs purified from the urine of control subjects (**a**, **b**) and diabetic patient (**d**, **e**). Size distributions of uEVs from control (**c**) and diabetic sample (**f**) were obtained by the NTA method.

Transmission electron microscopy images revealed the heterogeneity of the uEVs population in terms of size (50–300 nm) and electron density (Fig. 1a-e). Obtained electron density pattern of the uEVs membrane showed the integrity of the lipid bilayer and irregularity in the shape, which is typical for EVs isolated from human urine isolated by the filtration method [25]. The concentration (particles/ml) and size distributions of uEVs were determined by the NTA method. The mean diameter of uEVs originating from the urine of the control and the diabetic sample was equal to 226.7 ± 2.2 nm and 205.1 ± 0.9 nm, respectively (Fig. 1c, f).

### A Comprehensive Analysis of miRNAs in Human uEVs

**Fig. 2:**
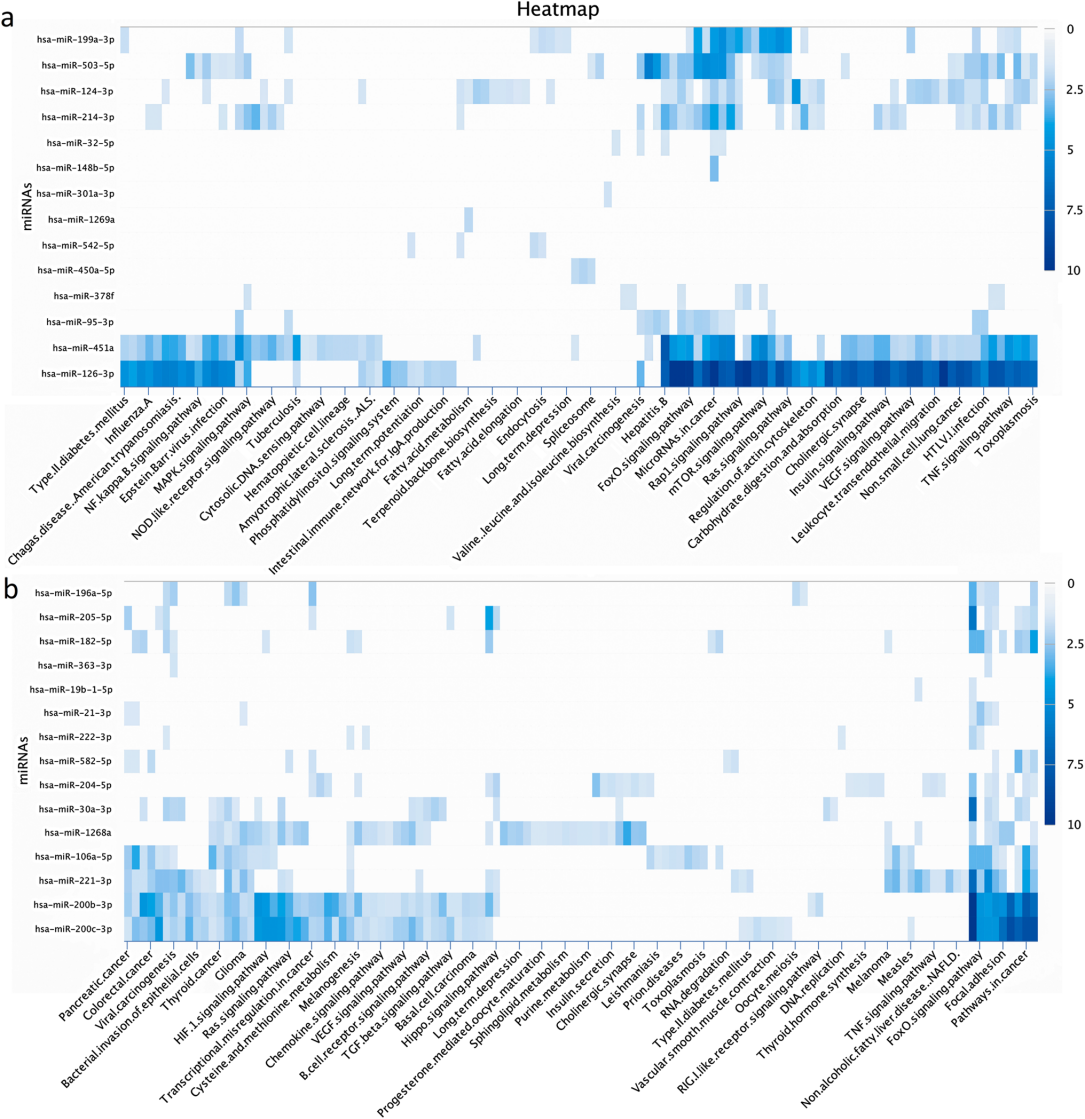
Heat maps (clustering based on significance levels) show KEGG pathways enriched in up-regulated (Fig. 2a) and down-regulated (Fig. 2b) miRNA of uEVs. Less intensive blue color indicates lower than mean intensity, and more intensive represents higher than mean intensity. Each row represents a miRNA, and each column represents a KEGG pathway. Heat maps were generated using DIANA mirPath v 3.0.

Comprehensive analysis of human uEVs miRNAs isolated from T2DM patients compared with healthy individuals revealed a diversity of miRNA sets. In the first step, the goal of the statistical analysis was to identify the unique miRNAs carried by uEVs from patients with CKD because of T2DM. In this step, the very set of 569 miRNAs was generated from the sequencing data. In addition, the predicted targets revealed from different sets demonstrated the regulatory action of miRNAs in biological processes. Based on the identified miRNAs, the grouping and clustering of the target genes was carried out, which enabled the selection of cell pathways potentially affected by a group of identified miRNAs. First, miRNA target genes were identified, followed by pathway analysis to reveal biological functions. Only pathways with a false discovery rate (FDR) < 0.05 were of interest. Accordingly, defined KEGG pathways enriched with the identified upregulated and down-regulated miRNAs are shown in Fig. 2.

**Fig. 3:**
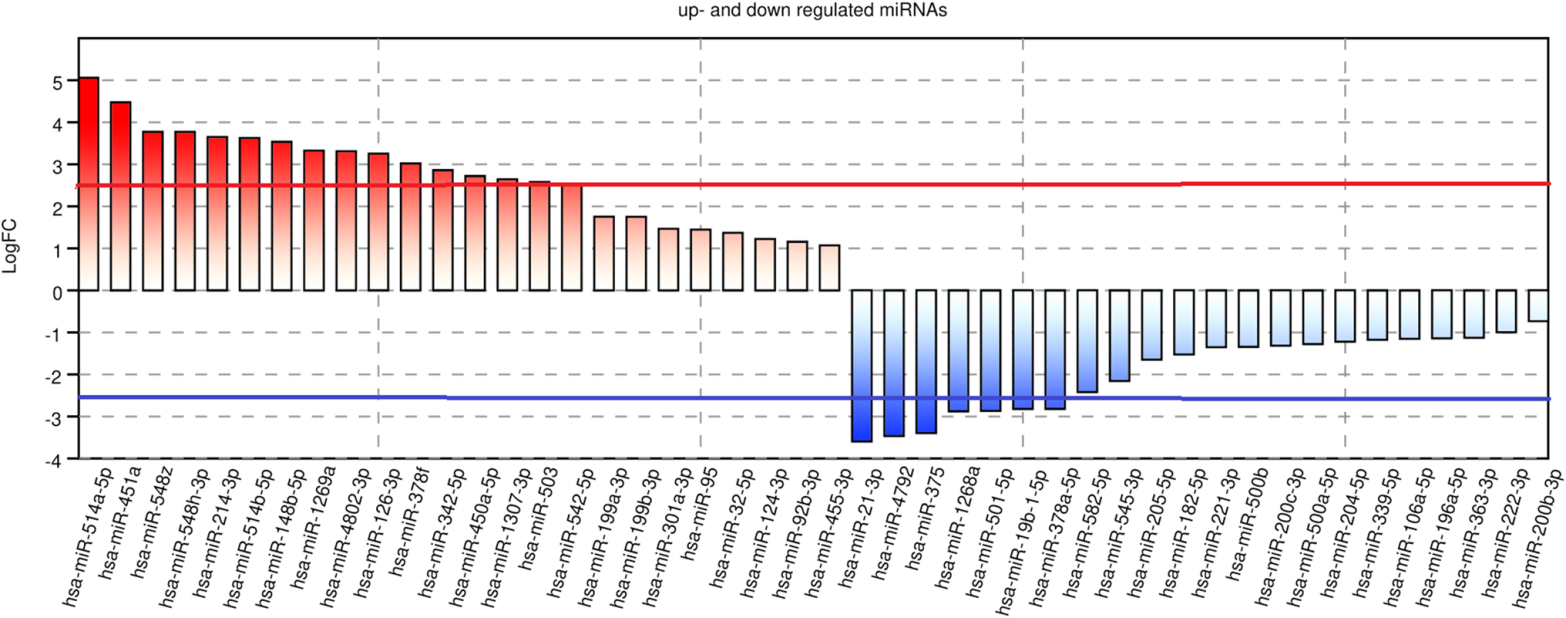
Different up-regulated—the top of the figure, and down-regulated – the bottom of this figure miRNAs. The logFC cutoff > 2,5 demonstrated the miRNAs with the most significantly different expression levels.

In the second step, each of these 569 miRNAs were checked to ensure that they were significantly enriched in functional categories. For each type of functionally category identified type, the number of significant associations with each miRNA was counted as shown in Table S1 (Supplementary File). Analysis of the miRNAs expression profiling dataset led to identifying 23 downregulated and 24 upregulated miRNAs, which were statistically significant in patients with DKD compared to healthy subjects. Among these miRNAs, 16 were significantly upregulated and 8 were downregulated. The cut-off for presenting data was a P-value < 0.005. These up- and down-regulated miRNAs identified were visualized in Fig. 3 and listed with their p-value, LogFC, and log CPM values in Table II.

**Table II:**
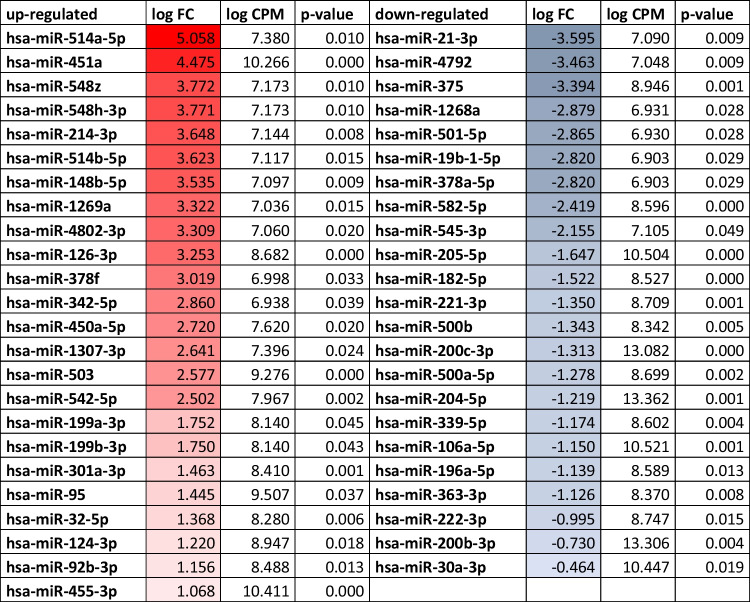
Differentially Expressed miRNAs were Selected Based on Statistically Significant Differences in Patients with DKD Compared to Healthy Subjects. A Positive Log Twofold-Change (logFC) Indicates Genes Up-Regulated in Diabetic Patients Relative to Healthy Controls, whereas a Negative logFC Indicates Genes More Highly Expressed in Healthy Controls. LogFC Values Relative to Upregulated miRNAs were Shown as a Gradient of Red Color in the Boxes, whereas a Rise of Blue Color in the Boxes was used for Downregulated miRNAs

*log CPM means the logarithm of counts per million reads

**Fig. 4:**
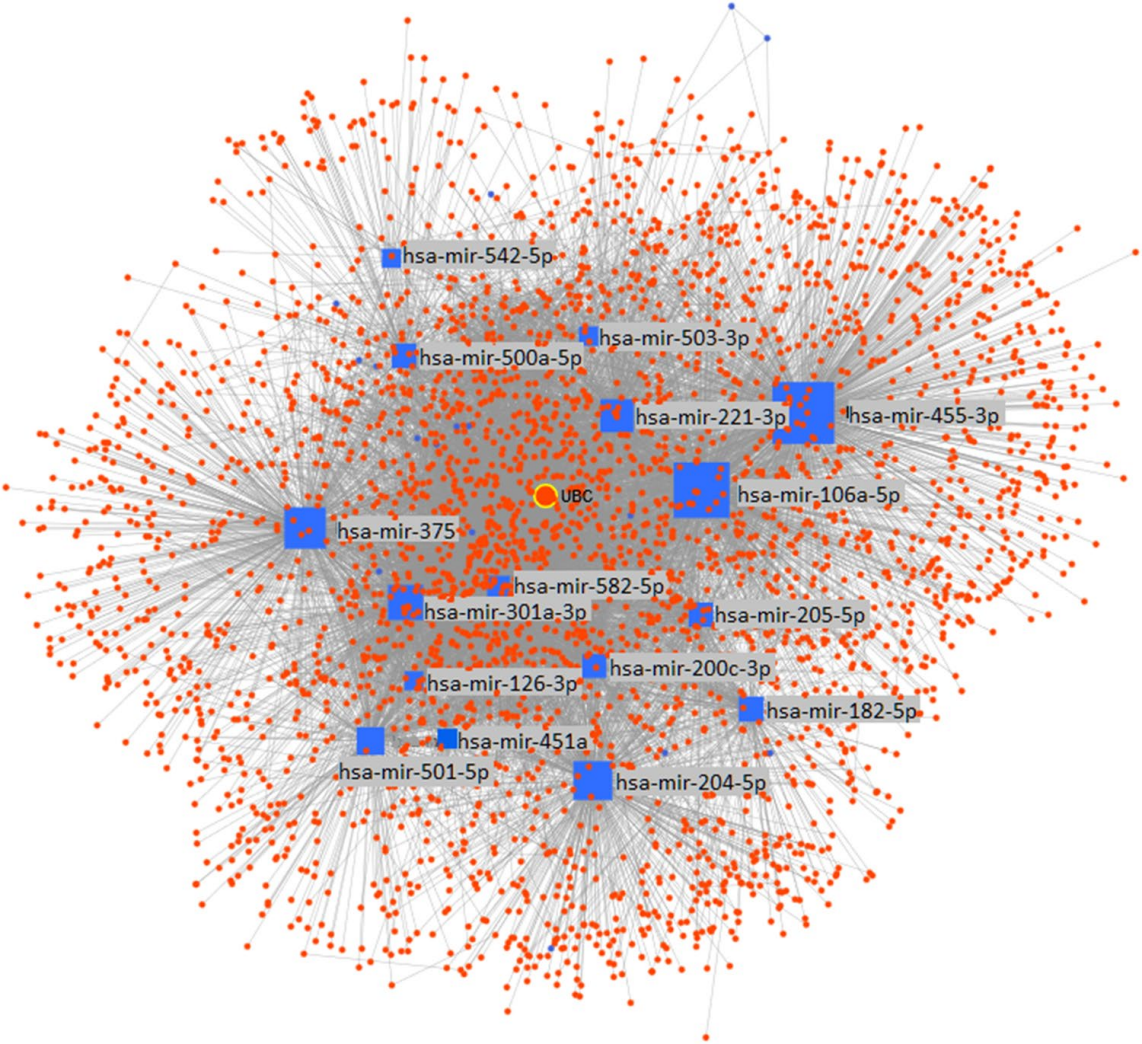
Genes—miRNAs regulatory networks. The blue squares are miRNAs, and the red spots are mRNA targets. The edge connecting two nodes is indicative of regulation. The more oversized shape indicates the highest target biological relevance of the miRNAs identified.

In the third step, up- and down-regulated miRNAs were annotated to the human miRNA-target interactions and miRNA-disease association data. This annotation was performed based on the Human MicroRNA Disease Database version 3.2 (HMDD v3.2). The results of the annotation are visualized in Fig. 4.

Then, the T2DM disease network with shared miRNAs was identified using HMDD and then compared with experimentally identified miRNAs. Target mRNAs for the miRNAs identified in this study were predicted using the MicroRNA Prediction Database (miRDB).

As shown in Fig. 4, hsa-miRr-106a-5p, hsa-miR-375, hsa-miR-451a, hsa-miR-500a-5p, hsa-miR-503, and hsa-miR-542-5p were associated with T2DM and other known, related diseases. The up-regulated hsa-miR-451a, down-regulated hsa-mirR-375, and up-regulated hsa-miR-503 seem to play a central role in regulating diabetes pathomechanism.

**Fig. 5:**
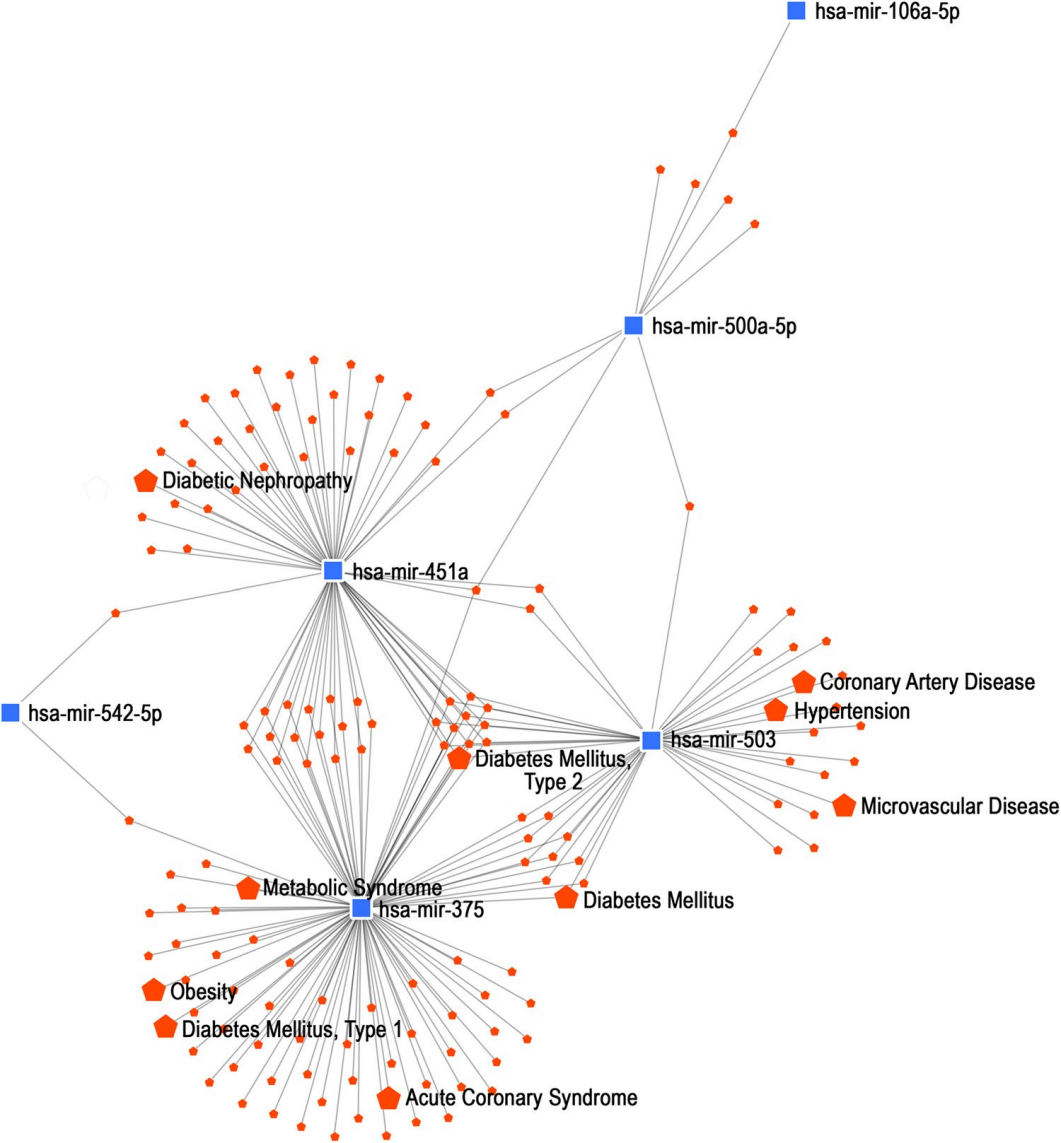
Up- and downregulated miRNAs associated with T2DM pathomechanism and other related diseases. The blue squares are miRNAs, and the red spots represent miRNAs-targets; red pentagons indicate pathomechanisms related to the disease.

**Fig. 6:**
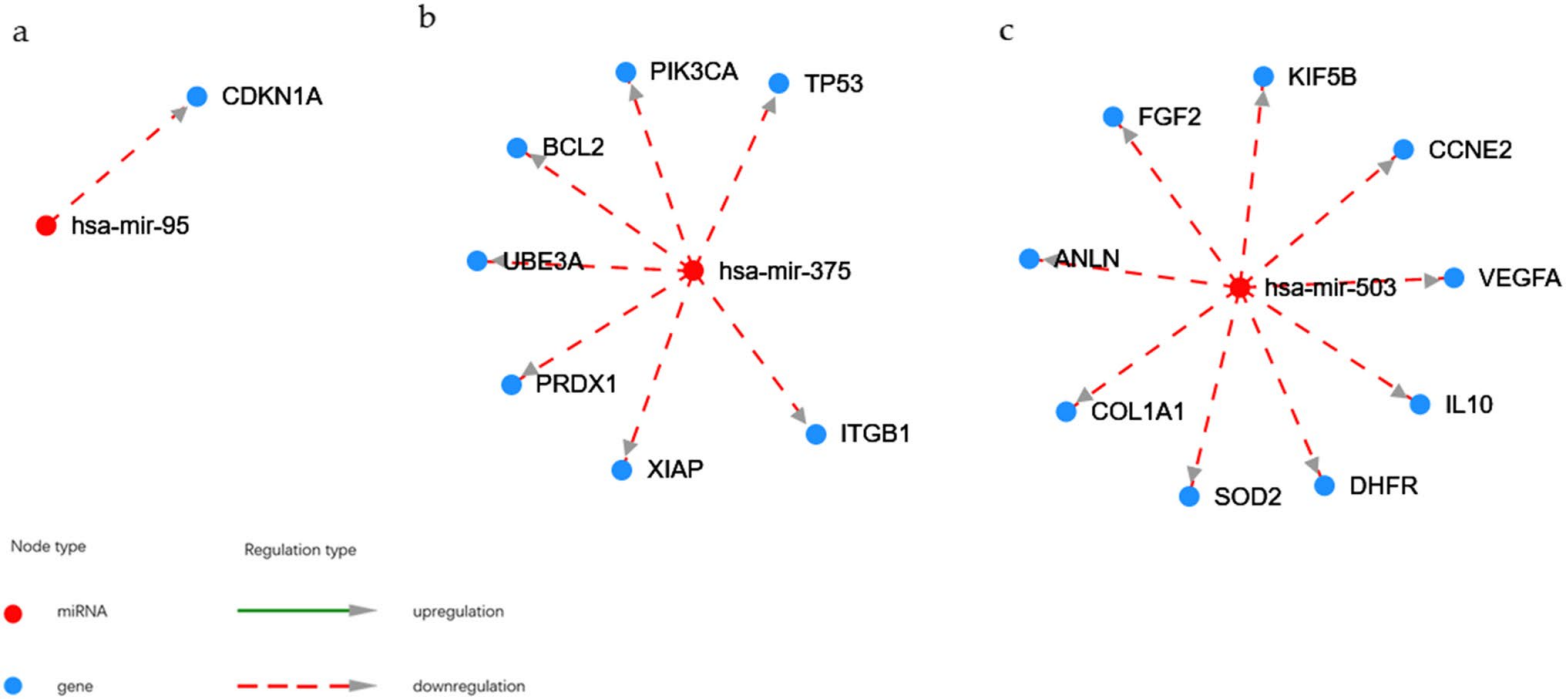
Target genes for hsa-miR-95 (Fig. 6a), hsa-miR-375 (Fig. 6b), and hsa-miR-503 (Fig. 6c).

The miRNAs that showed the highest association (based on logFC, p-value, and gene target interactions) with T2DM were identified and shown in Fig. 5. Two miRNAs, hsa-miR-451a, and hsa-miR-375 showed the strongest inter-individual interactions, and association with T2DM. For this reason, the target genes for these two miRNAs were checked, in the further step of the analysis, and are shown in Table III and in Fig. 6 are shown target genes for hsa-miR-95, hsa-miR-375, and hsa-miR-503 that we identified among these miRNAs which were strongly associated with T2DM.

**Table III:** The List of the Top Predicted Target Genes Regulated by hsa-mir-375 and hsa-miR-451a

miRNAs	Gene targets
hsa-miR-375 hsa-miR-451a	SPINK13, C3orf38, ELAVL4, COLCA2, KRTAP1-3, SERINC4, CR769776.1, ZIC1, SLC16A2, CLCA2, CACNG2, AC117395.1, EFCAB5, APEX1, POTEF, MS4A14, GREM2, RASD1, EIF4G3, MKRN1, DTHD1, EIF1AY, RPN1, XAF1, MMD, HIGD1A, ZFP36L2, LRAT, DIRAS3, POU3F1, NME5, SUPT3H, RP11-352D3.2, RANBP3, HOXD1, PRNT, MPC1, LRFN1, RLF, HOXB3, MSMP, WFDC10B, CXCL12, SOHLH2, MUC15, MXI1, PRDX1, ABCA10, PBRM1, STAM2, PIGL, TESPA1, CDH20, RP11-347C12.1, HNF1B, NPIPB11, CENPO, DIRAS2, NPY2R, DEFB114, TBC1D29, MTRNR2L13, ABRACL, NEK5, XKR6, HOXA5, CCL28, WBP1L, WFDC10A, NPIPB5, ITGB1BP2, PAPD4, SPOP, SEP15, ANKRD34A, ISL2, FUZ, AMOT, ZNF146, OR1I1, NPIPB4, FOXF1, DNMT3L, CLEC9A, ATP6V1G3, ZMAT4, ATPAF1, YBX1, SMIM18, PLEKHA3, CCDC169-SOHLH2, LDHB, RBPMS, ASF1A, CATSPER2, ZNF470, DCUN1D4, EBPL CUX2, PSMB8, CXCL16, TARP, ST8SIA4, CDKN2D, MIF, FBLN5, CERK, SAMD4B, CAB39, VAPA, LETM2, MEX3C, USP46, CMTM6, TBC1D9B, PMM2, KIAA1217, MAU2, RNF217, MEGF6, S1PR2, EVL, FBXO33, ATF2, CDKN2B, UCK1, CAV1, C16orf72, DCAF5, RAB5A, CACHD1, LUZP2, EIF2AK3, AKTIP, FAM171A1, TTN, NEDD9

In order to determine whether there are correlations between miRNAs determined in uEVs and epidemiological factors such as age, gender, presence or absence of diseases (T2DM), with biochemical parameters (serum lipids, glucose and creatinine, and others from Table I), statistical analyzes were performed taking these factors into account. The results of these analyzes are presented in Table S2 (Supplementary File). Statistically significant relationships were found between miRNA levels in uEVs and age (R = 0.636; p = 0.002), HbA1c (R = 0.693; p = 0.016), glucose (R = 0.765; p = 0.014) and the stage of disease (R = 0.960; p = 0).

The next stage of the analysis was to assess whether factors such as age and the occurrence of T2DM correlate with the expression levels of individual miRNAs. Due to the small number of groups, the analyzes were performed for the entire study population (Fig. S1). As a result of this analysis, the following miRNAs were found to negatively and significantly correlate with age: hsa-let-7e-3p; hsa-miR-107; hsa-miR-10a-3p; hsa-miR-16–2-3p; hsa-miR-200b-5p; hsa-miR-203; hsa-miR-30a-3p; hsa-miR-502-5p; hsa-miR-527; hsa-miR-659-3p (Table S3). Importantly, in the study group, the lowest levels of these miRNAs were observed in older patients (Figure S1).

Age-correlation analysis of those miRNAs that differentiated T2DM disease by up- and down-regulations, respectively (Fig. S2; Table S4), showed that only a few of them correlated with patients' age, such as: has-miR-659-3p; has-miR-502-5p; has-miR-338-5p; has-miR-203 (Table S4). When another discriminant, i.e. patient gender, was taken into account in this analysis, the number of correlated miRNAs increased to 8: hsa-miR-659-3p; hsa-miR-527; hsa-miR-502-5p; hsa-miR-500b; hsa-miR-500a-5p; hsa-miR-5009-3p; hsa-miR-362-5p; hsa-miR-16–2-3p, with 6 new miRNAs emerging (Fig. S3).

**Fig. 7:**
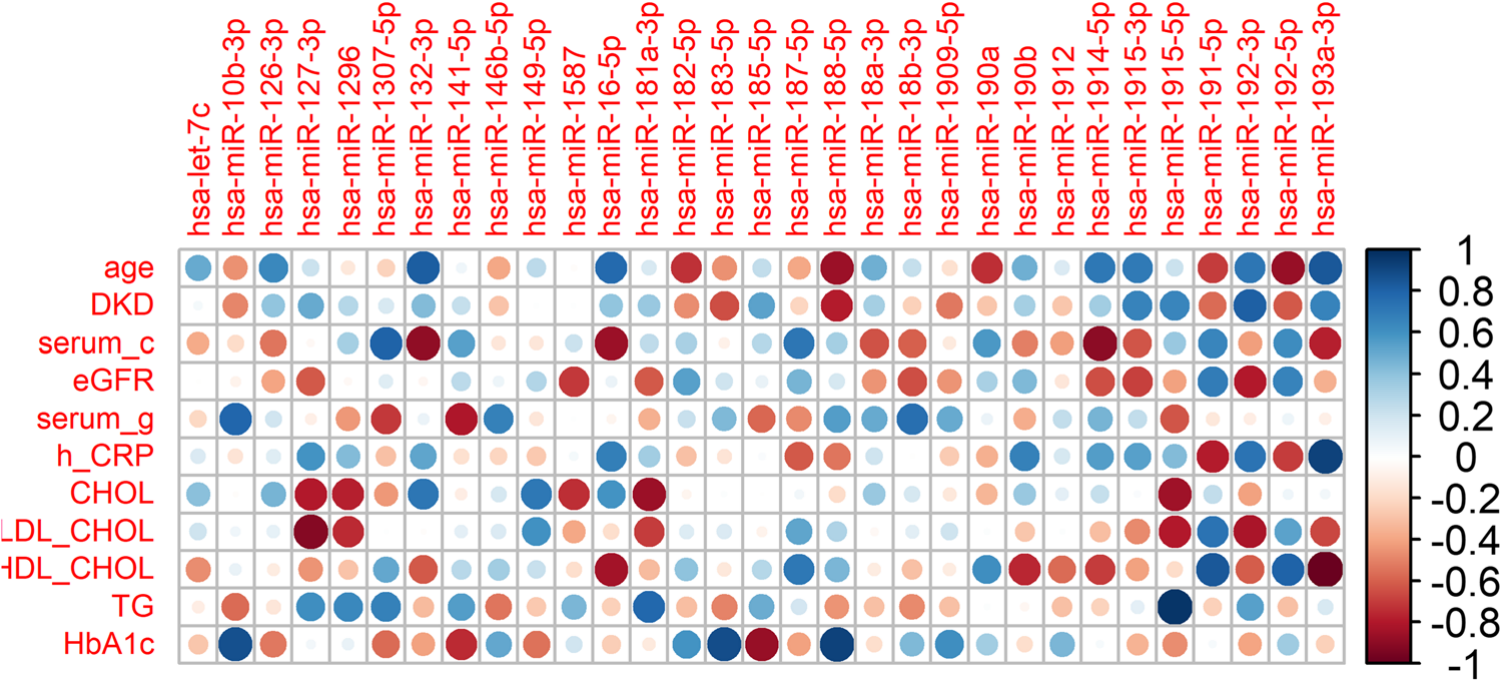
Heat map showing the correlation coefficients for miRNAs. Significant correlations are colored either in red (negative) or blue (positive) dots, while correlations that were not significant are shown as empty squares.

The last step of statistical analysis revealed a correlation between the identified miRNAs and biochemical parameters. The positive correlation between triglycerides (TG) and hsa-miR-1915-5p was most evident (close to 1). A similarly high correlation was found between hCRP and hsa-miR-193a-3p and be-tween HbA1c and hsa-miR-188-5p, hsa-miR-10b-3p, as shown in Fig. 7. The most pronounced negative correlations were observed for the following HDL CHOL pairs and hsa-miR-193a-3p, LDL_CHOL and hsa-miR-127-3p, serum creatinine (serum_c) and hsa-miR-132-3p, hsa-miR-16-5p, hsa-miR-1914-5p, as shown in Fig. 7. Among the miRNAs that correlated either positively or negatively with patients' biochemical characteristics, only hsa-miR-126-3p was reported as a diabetes-associated miRNA (according to miRTarBase). Hsa-miR-126-3p showed a significant positive correlation with age and total cholesterol level and negative correlation with serum creatinine, eGFR and HbA1c. For these reasons, hsa-miR-126-3p was further correlated with other miRNAs. Table IV shows the positive and one negative (with hsa-miR-1297) correlation of hsa-mi126-3p.

**Table IV:** The Correlation Coefficients Values for hsa-miR-126-3p and other miRNAs

	hsa-miR-126-3p
hsa-miR-18a-5p	0.847
hsa-miR-1294	0.847
hsa-miR-1200	0.847
hsa-miR-1202	0.841
hsa-miR-129-5p	0.832
hsa-miR-144-3p	0.827
hsa-let-7f-5p	0.825
hsa-miR-136-3p	0.824
hsa-miR-129–2-3p	0.810
hsa-miR-1185–1-3p	0.809
hsa-miR-1203	0.786
hsa-miR-1249	0.776
hsa-miR-187-5p	0.766
hsa-miR-18b-3p	0.765
hsa-miR-139-5p	0.765
hsa-miR-1206	0.760
hsa-miR-181d	0.758
hsa-miR-1915-5p	0.757
hsa-miR-1227	0.756
hsa-miR-143-3p	0.751
hsa-miR-1238	0.731
hsa-miR-1306-5p	0.7254
hsa-miR-1324	0.718
hsa-miR-1271-3p	0.707
hsa-miR-188-5p	0.697
hsa-miR-1297	-0.730

## Discussion

It has been previously reported that in patients with end-stage kidney disease treated with peritoneal dialysis (PD), there were differences in the proteome of PD effluent-containing EVs and urinary EVs. Therefore, EVs have been suggested as potential biomarkers of functional insufficiency of the peritoneal membrane [26, 27]. There are also many other reports emphasizing the role of EVs as paracrine factors involved in the mechanisms of cell-to-cell communication mechanisms in both acute and chronic kidney injury [28]. There is growing interest in using miRNAs transmitted by EVs from the urinary system as biomarkers of urinary tract diseases, including diabetic nephropathy or even rare renal tubular epithelia transporter disorders, is of [29–31].

EVs are believed to enhance activity of soluble factors, which, as a release from their parental cells, further potentiate their roles by transferring functional molecules such as miRNAs, mRNAs, and proteins to target cells, to demonstrate renoprotctive effects in the model of DKD [32, 33].

In this study, we examined the profile of miRNAs transferred by uEVs in patients suffering from complications of T2DM. In particular, we analyzed the potential contribution of identified miRNAs to DKD using bioinformatics tools.

Firstly, our analysis revealed significant data for the upregulated and downregulated specific miRNAs shown in Table II, amongst them, downregulated hsa-mir-375 and upregulated hsa-miR-503, hsa-miR-451a were reported to be linked and to play very important roles as therapeutic targets or biomarkers for T2DM, diabetes mellitus, and diabetic nephropathy. Human hsa-miR-375 is involved in the regulation of insulin secretion and it has been suggested that it may therefore be a new pharmacological target for the treatment of diabetes mellitus [34]. According to the HMDD data, hsa-miR-375 controls the expression of genes involved in apoptosis (TP53, BCL2, XIAP), cell protection (PRDX1), or protein degradation system (UBE3A) (Fig. 7). Other findings support the important role of hsa-miR-375 in regulating the phenotype of human β-cell, suggesting that the upregulation of hsa-miR-375 may facilitate the generation of functional insulin-producing cells following an *ex-vivo* expansion of human islet cells [35]. Haifa Abdullah Al-Muhtaresh and Ghada Al-Kafaji reported that both hsa-miR-and hsa-miR-9 were associated with the susceptibility to T2DM development and that hsa-miR-375 itself could serve as biomarkers for early detection of prediabetes and T2DM [36] and a sensor of glucotoxicity in children with newly diagnosed type 1 diabetes [37].

Additionally, to downregulated miRNAs, several studies also described the role of miRNAs, that were found to be upregulated in our study, notably hsa-mir-503, hsa-mir-451a, and hsa-mir-95. It was shown that deregulation of hsa-miR-503 influences T2DM-induced impairment of endothelial function and reparative angiogenesis after limb ischemia [38]. Compared to our study, the authors noticed that the hsa-miR-503 expression was remarkably higher, and plasma hsa-miR-503 levels were also elevated in diabetic individuals, suggesting its utility as a possible therapeutic target in diabetic patients with critical limb ischemia [32]. Xu *et al*. showed that hsa-miR-503 was upregulated in patients with gestational diabetes mellitus (GDM) and that its expression was positively correlated with blood glucose concentration [39]. They showed that has-miR-503 plays a key role in regulating pancreatic β-cell function by directly targeting the mTOR signaling pathway. These authors describhashsa-miR-503 as a potential therapeutic target in GDM [33]. Morehasr, hsa-miR-503, detected in serum, was also an effective tool for differentiating obese patients from those with T2DM [40]. The role of hsa-miR-503 in pathomechanism of diabetes complications depends on mitochondrial activity (DHFR), cell cycle control (CCNE2), cell protection (SOD2), podocyte migration (ANLN), inflammatory process (IL10, EFE2, VEGFA) and fibrosis (COL1A1), showing its contribution to CKD development. Known target genes for hsa-miR-451a were shown in Table III to better illustrate the possible involvement of this miRNA in biological processes. hsa-miR-451a. This micRNA was shown as a regulator factor for the p38 MAPK signaling pathway by targeting Ywhaz and suppressor factor in the mesangial hypertrophy in early diabetic nephropathies [40]. The hsa-miR-451a was also discovered as a down-regulated miRNA in patients with dilated cardiomyopathy, which facilitated the activation and proliferation of CD4 + T cells by targeting Myc [41].

Secondly, we did not observe significant upregulation of these ectosome-associated miRNAs, such as hsa-miR-95, hsa-miR-30, and hsa-miR-199, which have been already described as being strongly associated with the mechanisms underlying the development of vascular complications due to impaired angiogenesis in T2DM [16, 42]. The hsa-miR-95 is an suppressor of CDKN1A, and its down-regulation inhibits cell growth [42]. Another potentially involved in regenerating processes is has-miR-106a-5p, which was also found to be down-regulated in the study (Table II). It was also confirmed that overexpression of hsa-miR-199a-3p inhibits the proliferation, migration, and invasion of endothelial cells and retinal pericytes of diabetic retinopathy rats through regulating FGF7 via the EGFR/PI3K/AKT pathway [43]. It has been shown that EVs released from albumin-induced tubular EC promote macrophage proliferation [44]. Our study observed the enrichment of hsa-miR-199 in uEVs on the level below our discrimination criteria (logFC > 2.5), and hsa-miR-95 was even less upregulated.

Thirdly, very particular seemed to be the hsa-miR-451 which has already been described as a factor involved in p38 MAPK signaling by targeting the Ywhaz gene and suppressing the mesangial hypertrophy in early diabetic nephropathies [45]. Moreover, hsa-miR-451 has been previously reported and is known to play a vital role in early response to kidney cell damage. It was observed that the loss of hsa-miR-451 in proximal tubules resulted in the upregulation of the YWHAZ and CAB39 genes, which were suggested to provoke kidney fibrosis in humans [46]. Even so, low kidney hsa-miR-451 levels were correlated with kidney fibrosis in diabetic rats [47, 48].

Apart from the miRNAs mentioned above, hsa-miR-126-3p which has been upregulated in uEVs may also play a role in the development of vascular complications in T2DM, including nephropathy (Fig. 3). The hsa-miR-126-3p is known to maintain vascular integrity and contributes to vascular repair in hyperglycemia [49]. In the plasma of patients with diabetic nephropathy and in circulating exosomes in patients with T2DM, an increased level of hsa-miR-126-3p has been observed [16, 50].

In our study found the 3.6-fold increase of hsa-miR-214-3p in uEVs from DKD patients, selecting this miRNA as a potential candidate for a biomarker of DKD. It has been reported that upregulated hsa-miR-214-3p may promote the progression of kidney fibrosis in CKD [51], and a similar trend has been observed in hypertension, where T-cell–derived hsa-miR-214 controls pathological perivascular fibrosis [52].

At the end of this story, we would like to emphasize that for hsa-miR-514a-5p, we could not find the appropriate metabolic pathways in which this miRNA was involved. This miRNA was almost exclusively expressed in the testis samples, suggesting its tissue specificity [36, 53]. Known target genes for hsa-miR-514a are shown in Table III to better illustrate the possible involvement of the miRNAs in biological processes. Among these genes, metallopeptidases (ADAM10, MMP2, MMP9), inflammatory (IL6, IL6R, IKBKB, MIF), cell cycle and proliferation (CDKN2D, MYC) or apoptotic (BCL2) genes have their important position suggesting the role of this miRNA in fibrotic processes during CKD development. Additionally, the downregulation of hsa-miR-582-5p, which has not been reported so far in any diabetes-related pathways. This finding brings important shows that the pathomechanism of diabetic complications in kidneys is still not understood, and still is plenty of room for further research.

Concluding, miRNAs involved in highly regulated and key processes such as proliferation, differentiation, apoptosis, and metabolic pathways are dysregulated in the DKD. Our study takes note of the results of previous studies on the role of miRNAs in kidney metabolic homeostasis and shows that the imbalance caused by chronic T2DM is reflected in a different miRNA pattern in uEVs isolated from DKD patients [54]. Further research may elucidate the role of these miRNAs in regulating metabolic pathways during chronic kidney disease (CKD) progression and may select possible biomarkers of kidney injury in T2DM—DKD.

It is worth emphasizing that miRNAs may serve as potential therapeutic targets in metabolic disorders such as T2DM. Till now, many miRNAs have been detected in tissues and in serum and plasma, and other body fluids such as urine. It is essential to emphasize that these circulating miRNAs were seen in a stable form protected from endogenous RNase activity—EVs. EVs are involved in inflammatory and anti-inflammatory responses; thus, they can transfer both pro-and anti-inflammatory mediators [55]. As we have shown, the most enriched pathways involving the most significant deregulated miRNA are strongly related to inflammation (e.g., Immune System, Cytokine Signaling in Immune System, and the others shown in Table S1).

It means that uEV-related miRNAs may also be valuable biomarkers for complementary diagnosis. Our study is unique and concerns miRNAs detected in uEVs in patients with DKD as a complication of T2DM. Here we confirmed the role of known miRNAs. However, we also identified a few that are worth examining in a future study to ensure their involvement in the pathogenesis of T2DM and its consequences related to ***kidney*** failure.

## Materials and Methods

### Study Design

**Fig. 8:**
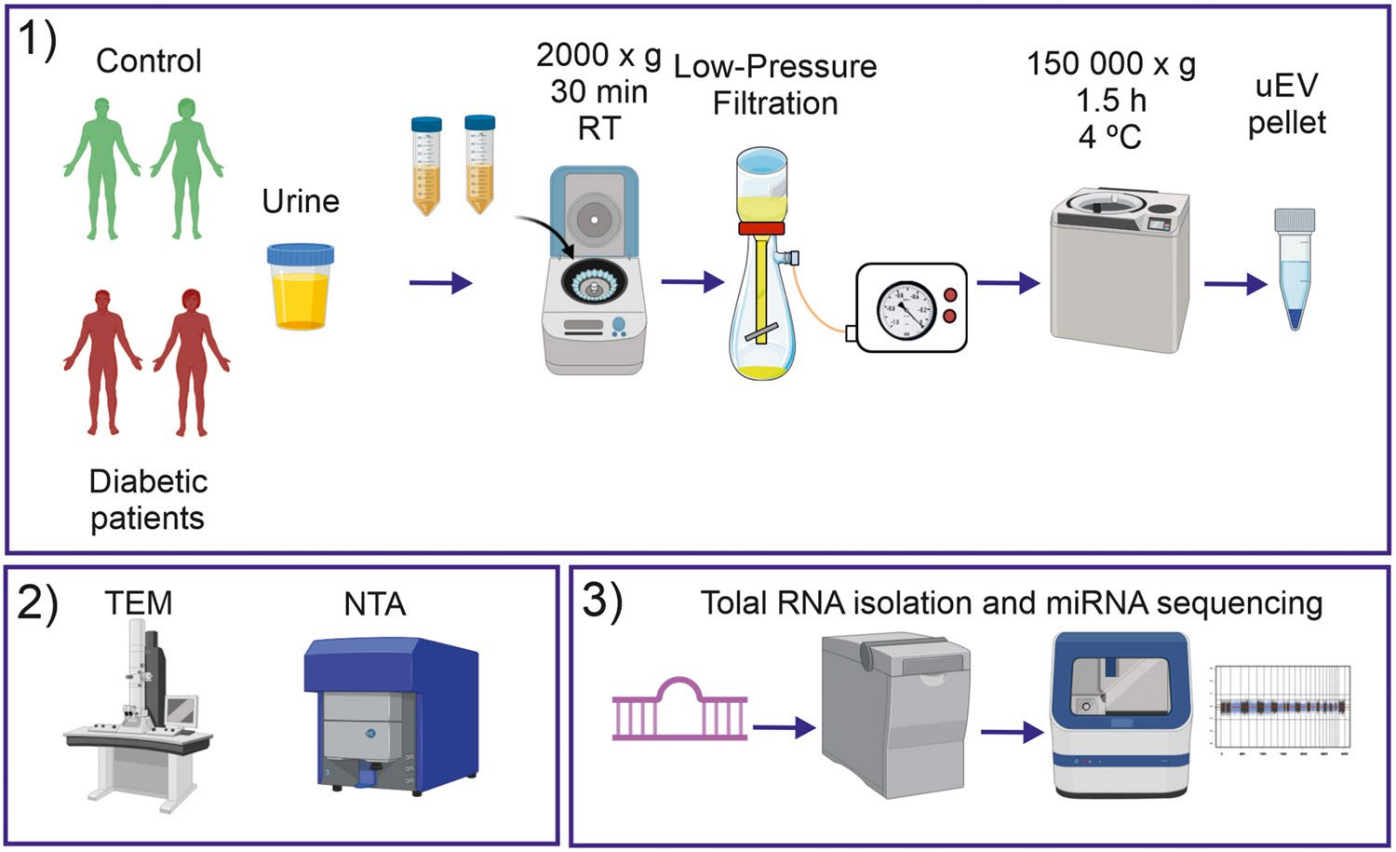
The scheme of the study design.

This was conducted at Queen Jadwiga Clinical District Hospital No2 in Rzeszów between 2018 and 2020. All patients with T2DM were diagnosed by a nephrologist. All subjects were invited to provide a blood sample for biochemical examination and urine samples for uEVs analysis. All protocols, procedures, and patient or control subjects’ recruitment were approved by the University of Rzeszów Bioethics Committee (permission no. 2018/06/16 from 14th June 2018). All participants provided written informed consent. Urine samples from patients with DKD and healthy subjects were used. The study design is illustrated in Fig. 8.

### Urine Samples Collection and Isolation of uEVs

First-morning mid-stream urine samples (50—80 ml) were obtained from eight diabetic patients with poor glycemic control and six healthy subjects. Urine samples were collected using sterile containers. EVs were isolated accordingly to Musante *et al*. [56] with slight modifications. Within 2 h after collection, urine samples were centrifuged in a Hermle Z300K for 30 min in 2000 g at room temperature (RT) to remove possible bacteria and cell debris. The supernatant was put into clean tubes. Next, sodium citrate (50 mM) and EDTA (8 mM) were added to the supernatant, and samples were frozen at –80 C until further processing. Before EVs isolation, urine samples were thawed at 37°C in a water bath. A colloidal silver solution composed of triclosan sodium (4.5 mg/1000 mL of urine) and colloidal silver (0.1 mg/1000 mL of urine) were added. The low-pressure filtration method was applied to isolate the fraction of uEVs using a cellulose membrane with a molecular weight cut-off (MWCO) equal to 1000 kDa [25]. After the initial sample concentration, the dialysis membrane was rinsed with 100 ml of deionized water to remove the dissolved urine fraction containing metabolites, salts, or free-floating proteins. After proper concentration of urine samples, approx. 1 ml of uEVs suspension was collected. Then the volume of uEV samples was normalized to the urine sample of the smallest volume. The total protein concentration in the uEVs samples was measured using a BCA protein assay kit (cat. No. 23227, Pierce BCA Protein Assay Kit, Thermo Fisher Scientific, MA, USA). The samples were used for further analysis.

### Transmission Electron Microscopy

Urinary EVs pellets isolated from control and diabetic urine sample were fixed with 2.5% GA (cat. no. G5882, Sigma-Aldrich, St. Louis, USA) in 0.1 M cacodylic buffer (Cat. number C4945, Aldrich, St. Louis, USA) for two hours at RT. Then, samples were postfixed in 1% osmium tetroxide solution (1 h) and dehydrated by passing through graded ethanol series and embedded in PolyBed 812 at 68°C. Ultrathin sections were collected on 300 mesh grids or one slot made from copper. Additionally, the latter was covered with formvar film. For cutting, the Leica EM UC7 microtome was used. Then, sections were contrasted using uranyl acetate and lead citrate. For observation, the JEOL JEM 2100HT electron microscope (Jeol Ltd, Tokyo, Japan) was used at an accelerating voltage of 80 kV.

### Nanoparticle Tracking Analysis

Urinary EVs samples were analyzed by the NanoSight NS300 Malvern system (Malvern Panalytical Ltd, UK) equipped with a 488 nm laser. Before measurement, the uEVs samples were diluted 2000 times in filtered (0.05 µm) deionized water. Measurements were performed at room temperature (25°C), and five videos for 60 s were recorded for UEVs samples. Data were captured with a camera detection threshold of 5 and syringe pump speed of 20 µl/min and analyzed using NTA 3.3 Build 3.3.104 software (NanoSight NS300, Malvern Instruments Ltd., Malvern, United Kingdom).

### RNA Isolation

Frozen pellets with uEVs from all urine samples were thawed on ice. All the uEVs samples were re-suspended in the PBS buffer. Exosomal RNA was isolated using the miRCURY RNA Isolation Kit (Exiqon, Vadbaek, Denmark) according to the manufacturer’s recommendations. All RNA samples were stored at -80 C for downstream applications. The quality, size, and concentration of the isolated RNA were determined using a capillary electrophoresis system with a total RNA 6000 Nano Chip and Small RNA chip following the manufacturer`s protocol (Agilent 2100 Bioanalyzer, Agilent Technologies, Santa Clara, CA, USA).

### miRNAs library construction and sequencing

The miRNAs libraries were prepared from the exosomal RNA using Xpress RNA-seq Barcode 1–16 Kit, Life Technologies, Carlsbad, CA, USA). For each library, 50 ng of RNA was used for ligation adapters with unique index barcodes. Furthermore, RNA samples were reverse transcribed to cDNA using adaptor-specific primers designed for small RNA sequencing. cDNA samples were then used for the size-selection step. Size selection from 94 to 200 nt that included the adaptor sequences around 25 bp was performed using Magnetic Bead Purification Module (Life Technologies). The yield and size distribution of the contracted small RNA libraries were verified using the Agilent 2100 Bioanalyzer instrument with the High Sensitivity DNA chip from Agilent. Only libraries with good quality were equally pooled. They were clonal amplified onto Ion Sphere Particles (ISPs) supplied by the Ion OneTouch 200 Template Kit v2 DL kit (Life Technologies) and enriched using the One Touch 2 ES System (Life Technologies).

### Sequencing Read Mapping and Minor RNA Annotation

Enriched ISPs were loaded with small RNA libraries and sequenced on the Ion Torrent PGM using Ion 318 V2 chips (Life Technologies) and the Ion PGM 200 V2 Sequencing Kit (Life Technologies). Two libraries were pooled and loaded per chip. A total of 1,819,299 mRNA sequence reads were generated through NGS sequencing. 1,685,283 were mapped, and 134,016 were unmapped. Data quality assessment was performed with a score lower than 30 on the PHRED scale. The quality of the next-generation sequencing data was evaluated automatically and manually. Reads from all passing samples were adapter trimmed and quality filtered using cutadapt v2.3 and filtered for a minimum length of 17 nt. The sequences were exported to the Genboree Workbench’s exeRtpt small RNA-seq pipeline (version 4.6.2). Output files were mapped to the Human genome HG19. Reads were mapped first against the genomic reference GRCh38.p12 provided by Ensembl, allowing for two mismatches, and subsequently miRBase v 22.1., filtered for microRNAs of hsa only, allowing for one mismatch. For a general RNA composition overview, non-microRNA mapped reads were mapped against RNAcentral and then assigned to various RNA species of interest. Reads were normalized to reads per million reads (RPM). To assess the functions, roles, and biological processes of the identified miRNAs and their enrichment in different biological pathways, a comprehensive functional annotation of miRNAs using miRNA was performed [27, 28]. A set of miRBase IDs selected based on previous calculations was used. The database of human data was searched, and links between the assigned miRNAs and genes and disease entities were found. For better visualization, miRNAs-targets miRNAs-target interaction networks were optimized using the Fruchterman-Reingold algorithm..

### Real-Time RT-PCR Quantification

To validate identified miRNAs with statistical significance, qPCR analysis of selected miRNA targets was performed. Applied Biological Materials Inc. (ABM, BC, Canada). First, 50 ng of exosomal total RNA was reverse transcribed with the miRNA cDNA synthesis kit, with Poly (A) polymerase tailing (ABM, BC, Canada) according to the manufacturer’s instructions. Then, equal amounts of cDNA were used for all the qPCR reactions. qPCR analysis was conducted with an Applied Biosystems 7900HT thermal cycler using the Fast Advanced Master Mix (Applied Biosystems). For 351 miRNAs, a large transit analysis was performed, and then for a selected few of miRNAs (such as hsa-miR-106a-5p, hsa-miR-126-3p, hsa-miR-182-5p, hsa-miR-200c-3p, hsa-miR-204-5p, hsa-miR-205-5p, hsa-miR-221-3p, hsa-miR-301a-3p, hsa-miR-375, hsa-miR-451a, hsa-miR-455-3p, hsa-miR-500a-5p, hsa-miR-503, hsa-miR-542-5p, hsa-miR-582-5p) the TaqMan Advanced miRNA Assays were used. The levels of differentially expressed miRNAs isolated from uEVs from patients with diabetic nephropathy were compared to the control subjects. The miRNA expression fold change (FC) was expressed as base-2 logarithm of FC (log2FC) to normalize the miRNA expression values.

### Statistics

RNA-seq FASTQ files were preprocessed, and their quality control was performed. Low-quality data was removed. An integrative statistical hypothesis testing method was performed, and all miRNAs in samples from study and control groups were compared using a t-test to define differentially expressed miRNAs. Multiple testing corrections were done using the Benjamini–Hochberg method [57]. A false discovery rate (FDR) less than 0.05 and absolute log fold change (logFC) greater than two were set as the significant cut-offs. The expression levels of the miRNAs were evaluated using the comparative CT method (2^−∆∆Ct^) in qRT-PCR. The hypothetical pathways analysis between the miRNAs and their mRNA targets was drawn by adopting the miRNA-mRNA target database of miRNet. Correlation analysis of miRNAs expression with age and biochemical parameters was performed using the Qlucore Omics Explorer tools (https://qlucore.com/omics-explorer).

## The Limitations of the Study

The main limitation of our study is the size of the study groups, the number of T2DM patients was thirteen and the number of healthy was six, which could affect the statistical significance of poorly expressed molecular parameters, i.e. some miRNAs. However, the main biochemical characteristics associated with diabetes and chronic kidney disease (eGFR) were significantly different (Table I), which means that the groups were correctly selected to prove the research hypothesis that miRNA contained in uEVs contribute to T2DM-CKD pathomechanism. The effect of gender bias also needs to be considered, as our study groups were not analyzed with due consideration for risk factor. Another aspect to consider is the heterogeneity within a study group. The median HbA1C level in patients enrolled in the present study was 10.1% (7.3%–14%), which shows wide variability and indicates impaired glucose regulation in T2DM patients enrolled in this study. However, the healthy controls included in the present study were not considerably different from those included in our previous studies [19, 24, 25].

Urinary content and concentration are variable and depend on many factors such as age, gender, diet, exercise, or medication taken. A large age variation within the group of diabetic patients (45–88) and a difference in average age between the control group (median = 50) and patients (median = 82.5) may also affect the physicochemical properties of EVs. In our previous study, we observed age has a stronger contribution in patients with CKD compared to patients without kidney disease [58]. In this study, the strong correlation of age with selected miRNAs was demonstrated.

The high diversity of EVs can hamper compatible analysis of miRNA in clinical samples. To reduce the sample preparation bias, we applied an approach to analyze whole EVs isolated from the urine sample (all EV subpopulations) [56]. We used the cellulose dialysis membrane with 1000 kDa MWCO to concentrate EVs from the urine sample, nevertheless the urinary EV sample may be slightly contaminated by Tamm Horsfall Protein (about 4% is retained), which has been visualized in our previous study using SEM imaging methods [59]. It has been described in previous articles that the applied urinary EV isolation method may also affect the yields of miRNAs [58]. After the low-pressure filtration procedure, the isolated uEV samples were frozen at -80°C. It has been proved that storage at -80°C and freeze–thaw cycles reduces EV concentration, purity and increases median particle size [60]. To prevent this, we reduced the storage time and avoided sample thawing.

## Conclusions

In conclusion, in the present study, we described a set of deregulated miRNAs in uEVs from patients with chronic ***kidney*** failure due to T2DM (DKD). Several of these miRNAs, especially hsa-miR-514a-5p, are worth investigating in the context of prognostic significance and should be validated in the future as potential therapeutic targets. This study also shows the abundance and diversity of miRNA-enriched EVs in the urine of diabetic patients. We cannot exclude that some of these EVs can have a regenerative potential, especially those containing miRNAs associated with proliferation or apoptosis inhibition, such as hsa-miR-95 or has-miR-106a-5p.

## Supplementary Information

Below is the link to the electronic supplementary material.

Supplementary file1 (DOC 380 KB)

## Data Availability

The datasets used and analysed during the current study will be available from the corresponding author on reasonable request.
