# Supplementary material for: miRNA Signature of Urine Extracellular Vesicles Shows the Involvement of Inflammatory and Apoptotic Processes in Diabetic Chronic Kidney Disease
Source: Pharm Res. 2023 Mar 1;40(4):817–32. doi: 10.1007/s11095-023-03481-5 (PMC10126023; doi:10.1007/s11095-023-03481-5)

miRNA signature of urine extracellular vesicles shows the involvement of inflammatory and apoptotic processes in diabetic chronic kidney disease

Barbara Zapała^1^, Agnieszka Kamińska^2^, Monika Piwowar^3^, Agnieszka Paziewska^4,5^, Agnieszka Gala-Błądzińska^6,7^, Ewa Ł. Stępień^2,8,9^*****

^1^ Department of Clinical Biochemistry, Jagiellonian University Medical College, Krakow, Poland; [barbara.zapala@uj.edu.pl](mailto:barbara.zapala@uj.edu.pl)

^2^ Department of Medical Physics, M. Smoluchowski Institute of Physics, Faculty of Physics, Astronomy and Applied Computer Science, Jagiellonian University, 30-348 Kraków, Poland; [agnieszka1.kaminska@uj.edu.pl](mailto:agnieszka1.kaminska@uj.edu.pl); e.stepien@uj.edu.pl

^3^ Department of Bioinformatics and Telemedicine, Jagiellonian University Medical College, Krakow, Poland; [monika.piwowar@uj.edu.pl](mailto:monika.piwowar@uj.edu.pl)

^4^ Department of Neuroendocrinology, Centre of Postgraduate Medical Education, Warsaw, Poland; ^5^ Institute of Health Sciences, Faculty of Medical and Health Sciences, Siedlce University of Natural Sciences and Humanities, Siedlce, Poland;

^6^ Medical College of Rzeszow University, Institute of Medical Sciences, Rzeszów, Poland; agala.edu@gmail.com

^7^ Department of Internal Medicine, Nephrology and Endocrinology, St. Queen Jadwiga Clinical District Hospital No2 in Rzeszów, Rzeszów, Poland

^8^ Total-Body Jagiellonian-PET Laboratory, Jagiellonian University, Kraków, Poland

^9^ Center for Theranostics, Jagiellonian University, Kraków, Poland

* Correspondence:

Ewa Stępień; [e.stepien@uj.edu.pl](mailto:e.stepien@uj.edu.pl) ;

Tel.: +48 12 664 4762

**Table S1.** Pathway analysis main findings based on 569 significantly different miRNAs isolated from urine extracellular vesicles (uEVs).

The number of significant associations (miRNAs targets) is counted as for each type of the functional category.

| **Pathways** | **miRNAs targets** | **P-value** |
| --- | --- | --- |
| BH3-only proteins associate with and inactivate anti-apoptotic BCL-2 members | 7 | 0.000102 |
| Signaling by TGF-beta Receptor Complex in Cancer | 7 | 0.000102 |
| Chk1/Chk2(Cds1) mediated inactivation of Cyclin B:Cdk1 complex | 9 | 7.85E-05 |
| G2/M DNA damage checkpoint | 11 | 4.28E-05 |
| Oxygen-dependent proline hydroxylation of Hypoxia-inducible Factor Alpha | 11 | 0.000207 |
| TGF-beta receptor signaling in EMT (epithelial to mesenchymal transition) | 12 | 4.46E-06 |
| Signaling by Leptin | 12 | 0.000255 |
| Pre-NOTCH Transcription and Translation | 13 | 8.91E-06 |
| Signal transduction by L1 | 13 | 4.40E-05 |
| Signaling by BMP | 13 | 0.000163 |
| VEGFR2 mediated vascular permeability | 13 | 0.000163 |
| SHC1 events in ERBB2 signaling | 13 | 0.000289 |
| Regulation of Hypoxia-inducible Factor (HIF) by oxygen | 14 | 0.000104 |
| Cellular response to hypoxia | 14 | 0.000104 |
| Activation of BH3-only proteins | 15 | 4.36E-06 |
| CD28 co-stimulation | 15 | 3.65E-05 |
| RHO GTPases activate PAKs | 16 | 5.47E-07 |
| SMAD2/SMAD3:SMAD4 heterotrimer regulates transcription | 16 | 2.35E-05 |
| RHO GTPases Activate WASPs and WAVEs | 16 | 7.37E-05 |
| VEGFR2 mediated cell proliferation | 17 | 2.02E-06 |
| Cyclin D associated events in G1 | 17 | 1.51E-05 |
| G1 Phase | 17 | 1.51E-05 |
| FCERI mediated MAPK activation | 19 | 3.15E-05 |
| Ca2+ pathway | 19 | 0.000124 |
| Oncogene Induced Senescence | 22 | 7.39E-10 |
| Intrinsic Pathway for Apoptosis | 23 | 2.01E-08 |
| Transcriptional activity of SMAD2/SMAD3:SMAD4 heterotrimer | 23 | 1.85E-06 |
| Regulation of actin dynamics for phagocytic cup formation | 24 | 2.96E-05 |
| Toll Like Receptor 10 (TLR10) Cascade | 27 | 0.000175 |
| Toll Like Receptor 5 (TLR5) Cascade | 27 | 0.000175 |
| MyD88 cascade initiated on plasma membrane | 27 | 0.000175 |
| ISG15 antiviral mechanism | 28 | 4.50E-05 |
| Antiviral mechanism by IFN-stimulated genes | 28 | 4.50E-05 |
| TRAF6 mediated induction of NFkB and MAP kinases upon TLR7/8 or 9 activation | 28 | 8.54E-05 |
| Toll Like Receptor 7/8 (TLR7/8) Cascade | 28 | 0.000116 |
| MyD88 dependent cascade initiated on endosome | 28 | 0.000116 |
| Toll Like Receptor 9 (TLR9) Cascade | 29 | 7.64E-05 |
| TRIF-mediated TLR3/TLR4 signaling | 31 | 6.07E-05 |
| MyD88-independent TLR3/TLR4 cascade | 31 | 8.12E-05 |
| Toll Like Receptor 3 (TLR3) Cascade | 31 | 8.12E-05 |
| Fcgamma receptor (FCGR) dependent phagocytosis | 34 | 3.45E-07 |
| PI3K/AKT Signaling in Cancer | 34 | 9.34E-06 |
| Activated TLR4 signalling | 35 | 0.000108 |
| Toll Like Receptor 4 (TLR4) Cascade | 35 | 0.000224 |
| Signaling by TGF-beta Receptor Complex | 36 | 2.41E-08 |
| RHO GTPases Activate Formins | 37 | 0.000169 |
| Signaling by Interleukins | 38 | 0.00023 |
| Platelet activation | 39 | 0.00006 |
| Oxidative Stress Induced Senescence | 44 | 6.64E-10 |
| PI3K events in ERBB4 signaling | 44 | 9.17E-09 |
| PIP3 activates AKT signaling | 44 | 9.17E-09 |
| PI3K events in ERBB2 signaling | 44 | 9.17E-09 |
| PI-3K cascade:FGFR1 | 44 | 9.17E-09 |
| PI-3K cascade:FGFR2 | 44 | 9.17E-09 |
| PI-3K cascade:FGFR3 | 44 | 9.17E-09 |
| PI-3K cascade:FGFR4 | 44 | 9.17E-09 |
| PI3K/AKT activation | 45 | 8.81E-09 |
| VEGFA-VEGFR2 Pathway | 46 | 2.71E-10 |
| GAB1 signalosome | 46 | 3.77E-09 |
| Signaling by VEGF | 47 | 2.41E-09 |
| Role of LAT2/NTAL/LAB on calcium mobilization | 47 | 7.99E-09 |
| Apoptosis | 51 | 9.54E-05 |
| Programmed Cell Death | 52 | 8.24E-05 |
| Downstream signaling events of B Cell Receptor (BCR) | 60 | 1.30E-06 |
| Downstream signaling of activated FGFR1 | 62 | 1.05E-10 |
| Downstream signaling of activated FGFR2 | 62 | 1.05E-10 |
| Downstream signaling of activated FGFR3 | 62 | 1.05E-10 |
| Downstream signaling of activated FGFR4 | 62 | 1.05E-10 |
| Signaling by ERBB4 | 62 | 4.38E-10 |
| Cellular Senescence | 63 | 1.40E-10 |
| Signaling by SCF-KIT | 64 | 7.80E-13 |
| Signaling by the B Cell Receptor (BCR) | 66 | 3.18E-06 |
| Signaling by FGFR | 67 | 2.43E-11 |
| Signaling by FGFR1 | 67 | 2.43E-11 |
| Signaling by FGFR2 | 67 | 2.43E-11 |
| Signaling by FGFR3 | 67 | 2.43E-11 |
| Signaling by FGFR4 | 67 | 2.43E-11 |
| DAP12 signaling | 67 | 7.09E-11 |
| Downstream signal transduction | 68 | 7.44E-12 |
| DAP12 interactions | 69 | 1.99E-09 |
| Signaling by ERBB2 | 70 | 9.59E-13 |
| Signaling by EGFR | 73 | 1.05E-11 |
| Generic Transcription Pathway | 73 | 6.53E-09 |
| Signaling by PDGF | 74 | 7.11E-11 |
| NGF signalling via TRKA from the plasma membrane | 78 | 4.62E-11 |
| Diseases of signal transduction | 78 | 3.10E-06 |
| Fc epsilon receptor (FCERI) signaling | 79 | 1.13E-14 |
| Cell Cycle | 84 | 1.21E-04 |
| RHO GTPase Effectors | 85 | 1.16E-08 |
| Cytokine Signaling in Immune system | 85 | 9.35E-05 |
| Signalling by NGF | 100 | 3.27E-10 |
| Cellular responses to stress | 103 | 2.18E-13 |
| Signaling by Rho GTPases | 108 | 1.93E-06 |
| Adaptive Immune System | 131 | 3.90E-07 |
| Hemostasis | 135 | 6.75E-07 |
| Cell Cycle | 147 | 6.04E-07 |
| Innate Immune System | 167 | 1.44E-07 |
| Disease | 178 | 5.09E-05 |
| Gene Expression | 250 | 4.70E-11 |
| Immune System | 266 | 9.70E-10 |

**Table S2.** Summary table presenting statistical methods and tests applied for correlation analysis, calculated parameters and significance of analysed data

Analysis was performed using the Qlucore Omics Explorer tool (<https://qlucore.com/omics-explorer>). To the analysis all miRNA-seq data were included.

| **Response Variable** | **Response Variable** | **Min.**  **p-value** | **Min.**  **q-value** | **Two Group** | **Two Group** | **Two Group** | **Two Group** | **Two Group** | **Two Group** |
| --- | --- | --- | --- | --- | --- | --- | --- | --- | --- |
| ***Type*** | ***Details*** |  |  | ***Disease [T2DM≠All]**** | ***Disease [T2DM≠All]**** | ***Disease [T2DM≠All]**** | ***Disease [T2DM≠All]**** | ***Disease [T2DM≠All]**** | ***Disease [T2DM≠All]**** |
|  |  |  |  | Test Statistic Type | Statistic  parameter | p-value | q-value | Difference | R-statistic |
| Linear | **Age** | 0.002 | **0.006** | t-statistic | **-4.151** | 0.044 | **0.006** | 20.597 | **0.636** |
| Linear | CHOL (mmol/l) | 0.488 | 0.558 | t-statistic | 0.72 | 0.566 | 0.558 | -0.525 | -0.208 |
| Linear | HDL-CHOL (mmol/l) | 0.83 | 0.885 | t-statistic | -0.22 | 0.755 | 0.885 | 0.078 | 0.115 |
| Linear | **HbA1c (%)** | 0.007 | **0.016** | t-statistic | **-3.378** | 0.023 | **0.016** | 6.991 | **0.693** |
| Linear | LDL-CHOL (mmol/l) | 0.423 | 0.52 | t-statistic | 0.836 | 0.540 | 0.52 | -0.581 | -0.235 |
| Linear | Serum creatininine (umol/l) | 0.195 | 0.312 | t-statistic | -1.39 | 0.367 | 0.312 | 119.175 | 0.360 |
| Linear | **Serum glucose (mmol/l)** | 0.005 | **0.014** | t-statistic | **-3.57** | 0.007 | **0.014** | 13.117 | **0.765** |
| Linear | **Stages of the disease** | 0 | 0 | t-statistic | **-10.944** | 0.000 | **0** | 2.364 | **0.960** |
| Linear | TG (mmol/l) | 0.259 | 0.376 | t-statistic | -1.198 | 0.486 | 0.376 | 0.518 | 0.274 |
| Linear | eGFR (ml/min/1.73cm2);  CKD EPI | 0.001 | **0.003** | t-statistic | **4.697** | 0.006 | **0.003** | -46.149 | **-0.788** |
| Linear | hCRP (mg/l) | 0.298 | 0.397 | t-statistic | -1.099 | 0.428 | 0.397 | 38.698 | 0.315 |
| Multi Group | Age groups | 0.016 | 0.028 | Deviance statistic | 8.318 | 0.367 | 0.028 |  |  |
| Two Group | Age groups 60 Women  and >65 man [I] | 0.01 | **0.019** | Deviance statistic | **6.695** | 0.050 | **0.019** |  |  |

Numbers showing significant correlations are bolded between analysed variable and expression of total miRNAs as number of normalised reads.

**Figure S1.** Correlations of miRNAs isolated from urine extracellular vesicles (uEVs) with age for all patients. Only significant miRNAs are presented.

Analysis was performed for the healthy control group (***green tringles***) and T2DM patients (***red tringles***).


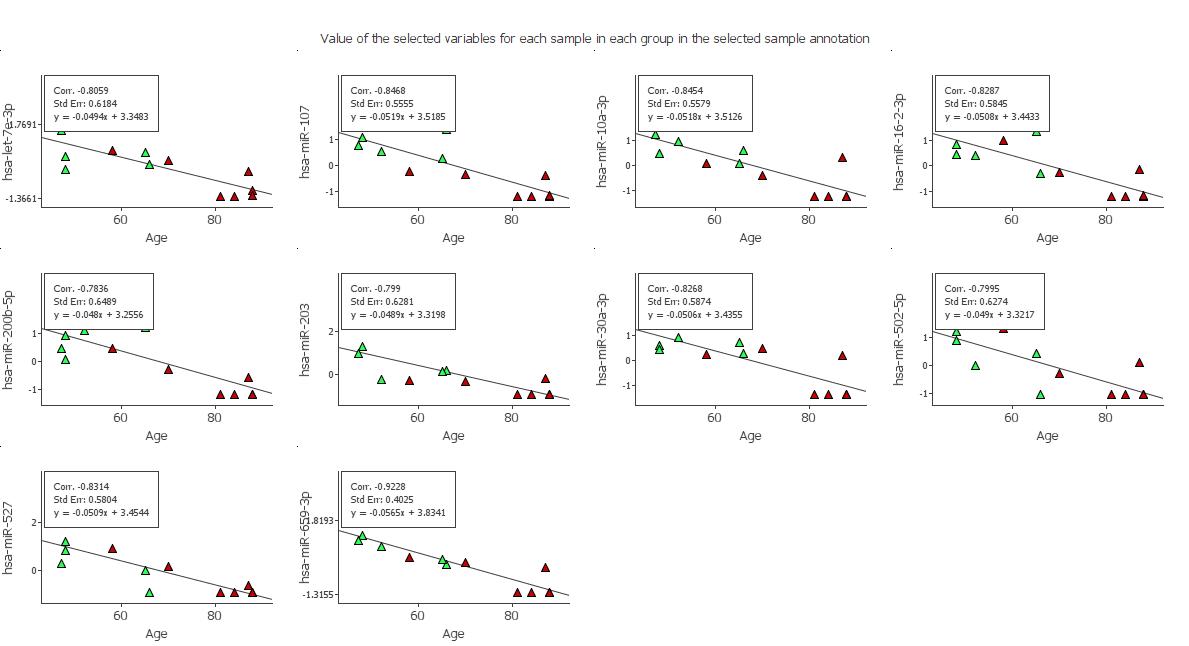


**Table S3.** Correlation coefficients for all miRNAs expressed in uEVs

miRNAs most significantly correlated with age were calculated for all patients in the study group.

| **miRNA** | **p-value** | **q-value** | **R statistic** |
| --- | --- | --- | --- |
| hsa-let-7e-3p | 0.001 | 0.202 | -0.806 |
| hsa-miR-107 | 0.000 | 0.185 | -0.847 |
| hsa-miR-10a-3p | 0.000 | 0.185 | -0.845 |
| hsa-miR-16-2-3p | 0.000 | 0.185 | -0.829 |
| hsa-miR-200b-5p | 0.002 | 0.202 | -0.784 |
| hsa-miR-203 | 0.001 | 0.202 | -0.799 |
| hsa-miR-30a-3p | 0.000 | 0.185 | -0.827 |
| hsa-miR-502-5p | 0.001 | 0.202 | -0.799 |
| hsa-miR-527 | 0.000 | 0.185 | -0.831 |
| hsa-miR-659-3p | 0.000 | 0.016 | -0.923 |

**Table S4.** Correlation coefficients with age and miRNAs isolated from uEVs.

The analysis was performed only for miRNAs distinguishing healthy people from T2DM patients. The list of correlated miRNAs is presented in Table 2 and Figure 3.

| **miRNA** | **p-value** | **q-value** | **R statistic** |
| --- | --- | --- | --- |
| hsa-miR-659-3p | 0.000 | 0.453 | -0.841 |
| hsa-miR-502-5p | 0.001 | 0.453 | -0.820 |
| hsa-miR-338-5p | 0.001 | 0.681 | -0.794 |
| hsa-miR-203 | 0.000 | 0.453 | -0.827 |

**Figure S2.** Correlations of miRNAs with age for all patients.

The analysis was performed only for miRNAs distinguishing healthy people from T2DM patients. Only significant miRNAs are presented. The study group was divided to three subgroups : patients between 40-49 years of age (***blue triangles***); between 50-59 years (***orange triangles***). while in the third >60 years of age (***yellow triangles***)


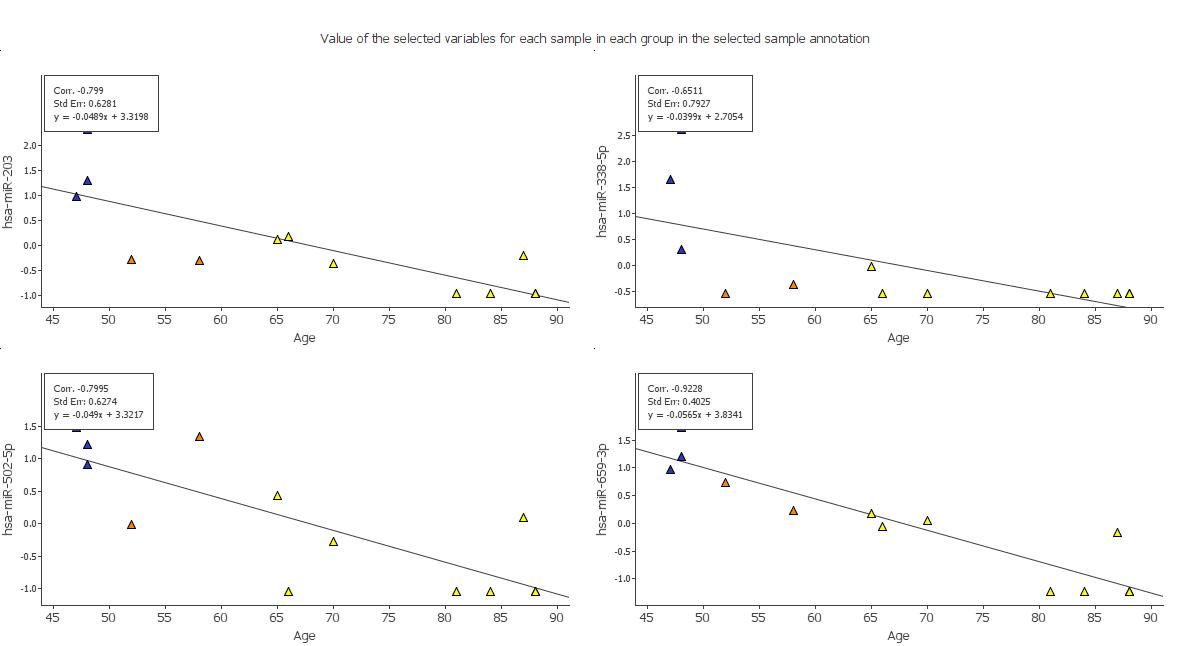


**Table S5.** Correlation coefficients miRNAs with age and gender of patients.

The analysis was performed only for miRNAs distinguishing healthy people from T2DM patients. The list of correlated miRNAs is presented in Table 2 and Figure 3.

The study group was divided into two age groups: women <60 years old and men <65 years old or women > 60 years old and men > 65 years old.

| **miRNA** | **p-value** | **q-value** | **R statistic** |
| --- | --- | --- | --- |
| hsa-miR-659-3p | 0.001 | 0.245 | -0.811 |
| hsa-miR-527 | 0.000 | 0.160 | -0.837 |
| hsa-miR-502-5p | 0.000 | 0.157 | -0.864 |
| hsa-miR-500b | 0.001 | 0.245 | -0.807 |
| hsa-miR-500a-5p | 0.000 | 0.160 | -0.842 |
| hsa-miR-5009-3p | 0.001 | 0.245 | -0.807 |
| hsa-miR-362-5p | 0.000 | 0.160 | -0.841 |
| hsa-miR-16-2-3p | 0.000 | 0.133 | -0.885 |

**Figure S3.** Correlation coefficients miRNAs with age and gender of patients.

The analysis was performed only for miRNAs distinguishing healthy people from T2DM patients as in the Table S4. The study group was divided to The patients were divided into two age groups: in the first group there were women <60 years old and men <65 years old (***dark blue triangles***). in the second group there were women > 60 years old and men > 65 years old (***light blue triangles***).


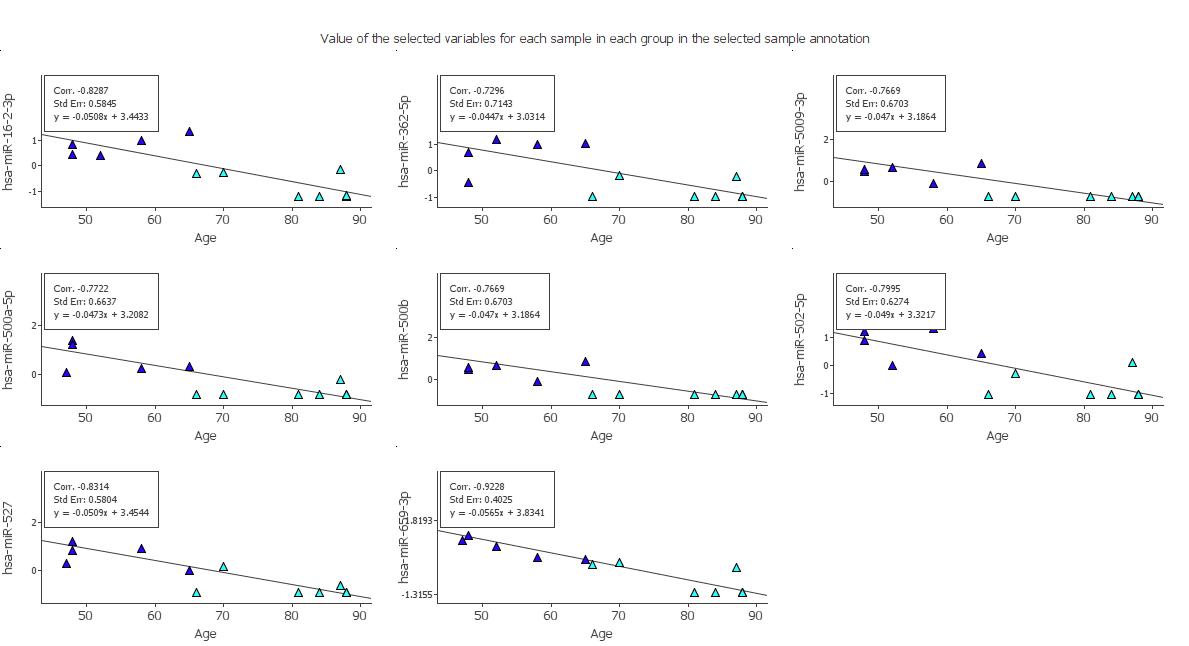

Supplement: Supplementary file 1 — Supplementary file1 (DOC 380 KB) [file 11095_2023_3481_MOESM1_ESM.doc]
